# Identification of Novel Noninvasive Diagnostics Biomarkers in the Parkinson's Diseases and Improving the Disease Classification Using Support Vector Machine

**DOI:** 10.1155/2022/5009892

**Published:** 2022-03-15

**Authors:** Shadi Moradi, Leili Tapak, Saeid Afshar

**Affiliations:** ^1^Department of Medical Immunology, School of Medicine, Hamadan University of Medical Science, Hamadan, Iran; ^2^Department of Biostatistics, School of Public Health, Modeling of Noncommunicable Diseases Research Center, Hamadan University of Medical Sciences, Hamadan, Iran; ^3^Research Center for Molecular Medicine, Hamadan University of Medical Sciences, Hamadan, Iran; ^4^Department of Molecular Medicine, School of Advanced Medical Sciences and Technologies, Hamadan University of Medical Sciences, Hamadan, Iran

## Abstract

**Background:**

Parkinson's disease (PD) is a neurological disorder that is marked by the deficit of neurons in the midbrain that changes motor and cognitive function. In the substantia nigra, the selective demise of dopamine-producing neurons was the main cause of this disease. The purpose of this research was to discover genes involved in PD development.

**Methods:**

In this study, the microarray dataset (GSE22491) provided by GEO was used for further analysis. The Limma package under R software was used to examine and assess gene expression and identify DEGs. The DAVID online tool was used to accomplish GO enrichment analysis and KEGG pathway for DEGs. Furthermore, the PPI network of these DEGs was depicted using the STRING database and analyzed through the Cytoscape to identify hub genes. Support vector machine (SVM) classifier was subsequently employed to predict the accuracy of genes.

**Result:**

PPI network consisted of 264 nodes as well as 502 edges was generated using the DEGs recognized from the Limma package under the R software. Moreover, three genes were identified as hubs: *GNB5*, *GNG11*, and *ELANE*. By using 3-gene combination, SVM found that prediction accuracy of 88% can be achieved.

**Conclusion:**

According to the findings of the study, the 3 hub genes *GNB5*, *GNG11*, and *ELANE* may be used as PD detection biomarkers. Moreover, the results obtained from SVM with high accuracy can be considered as PD biomarkers in further investigations.

## 1. Introduction

The dopaminergic neurons demise in the substantia nigra (SN) causes Parkinson's disease (PD) and ultimately induces severe and progressive motor impairment [1]. The detection of PD is made through a physical examination and motor symptoms evaluation such as bradykinesia, resting tremor, and muscle rigidity. Furthermore, years to decades before motor symptoms emerge, REM-behavior disorder, constipation, depression, and anosmia develop [2]. The change of soluble a-Syn aggregate particles to insoluble a-Syn aggregates is a crucial event in the pathogenesis of PD [3].

a-Syn forms accumulate in neurons, which can cause Lewy body pathology and dopaminergic neuron dysfunction [4]. Which causes neuroinflammation and impairs cellular activities such as autophagy, lysosomal and mitochondrial activities, microtubule transport, and vesicular homeostasis [5]. However, about 10% of PD cases are hereditary and cause early initiation. The majority of cases are idiopathic and linked to aging, environmental toxins, pesticides as well as heavy metals, painful lesions, viral, and bacterial infections, in addition to genetic susceptibility [6]. Furthermore, mitochondrial dysfunction can be linked to increasing the toxic oxygen species creation in PD, which has a role in inflammation [7]. In addition to astrogliosis and microgliosis in PD brains, PD-risk-associated genes and peripheral inflammation imply that the systemic inflammatory response is involved in the progression of this neurodegenerative disease [8]. Microglia split functionally in response to inflammatory stimuli into the M1 which is the proinflammatory phenotype or the cytoprotective and immunosuppressive M2 phenotype. [9]. Misfolded and aggregated proteins such as *α*-Syn, as well as signals from Toll-like receptors (TLRs), promote the development of the M1 proinflammatory microglial phenotype. There are only a few treatments available for PD right now. Levodopa, dopamine receptor agonists, adenosine 2A receptor antagonists, and apomorphine are some of the current PD treatments [10]. The delivery of l-DOPA as a source of DA is the foundation of contemporary DA (dopaminergic neuron) replacement therapy. L-DOPA which penetrates the blood-brain barrier is used as replacement therapy. In most patients, L-DOPA is initially useful in alleviating symptoms, but it loses effectiveness over many years of treatment [11].

While research suggests that molecular pathways such as mitochondrial function, inflammation, and oxidative stress play a role in PD pathogenesis, the exact pathogenic mechanisms of PD are still unknown. As a consequence, the main aim of this investigation is to use biological and bioinformatics system techniques to find new biomarkers that are relevant for PD [12].

## 2. Material and Method

### 2.1. Data Preprocessing and DEG Screening

Data on gene expression profiling with a series number GSE22491 based on the platform GPL6480 was recruited from the GEO database. This data comprises the gene expression levels of 8 control and 10 PD samples of peripheral blood mononuclear cells.

The Limma package in R was conducted to recognize the (differentially expressed genes) DEGs between PD samples and control samples. Adjusted *p* value less than 0.05 as well as log fold change more than 1 and less than -1 were picked up as splitting levels for DEGs.

### 2.2. Analyses of the DEGs' Functional Enrichment

In this research, Kyoto Encyclopedia of Genes and Genomes (KEGG) and Gene Ontology (GO) enrichment analysis were used to investigate the function of DEGs. Molecular function (MF), cellular component (CC), and biological processes (BP) are all part of the GO classification system. Database for Annotation Visualization and Integrated Discovery (DAVID) program [13] was used for enrichment analysis with fisher exact < 0.05 as the cut-off threshold.

### 2.3. PPI Network Integration

Protein-protein interaction (PPI) network of the DEGs was illustrated by use of the Search Tool for the Retrieval of Interacting Genes (STRING). In the current study, the interaction network was created by a confidence score of 0.7 as significant. The constructed network was visualized and analyzed with Cytoscape software. The CytoHubba package under Cytoscape software was conducted to evaluate the network and determine the hub genes through the MNC and MCC and Degree ranking methods.

### 2.4. Support Vector Machine (SVM)

The support vector machine (SVM) is a regression/classification method that is based on learning [14, 15]. Let us consider y as the binary response taking its labels as -1 and +1 (here, PD patients and healthy individuals) and *X* as the vector of p genes (gene expression profiling).

**Figure 1:**
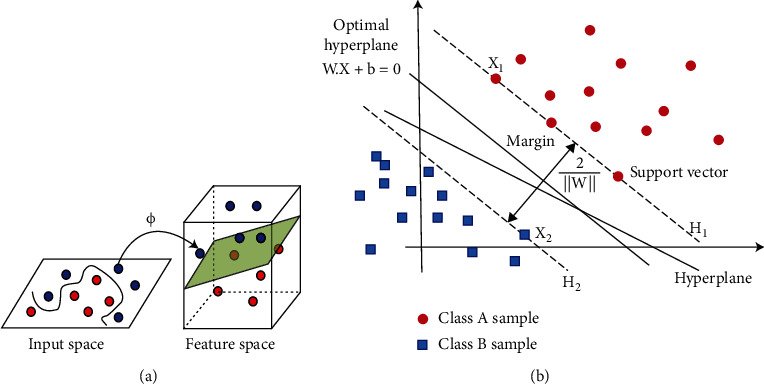
(a) The projection of input space to a space with a higher dimension in support vector machine. (b) Classification of data by support vector machine (SVM).

The main idea of the SVM is to use a kernel function to project the data in the feature space with a lower dimension (that may be nonlinearly separable) into points in a space with a higher dimension. Then, it separates the data into classes (e.g., patient/normal) using an optimal hyperplane by exploiting the principal of structural risk minimization (see Figure 1(a)) [16]. The equation of the linear hyperplane is as follows

(1) $$\begin{array}{c} w.x+b=0, \end{array}$$

and the margin 1/‖**w**‖ between two classes is maximized (where ‖**w**‖ is the norm of the coefficients vector **w**) (Figure 1(b)) [17]. Let us consider a *p*-variate vector of *x*_*j*_ ∈ ℝ^*p*^ (*j* = 1, ⋯, N) be the features related to the subject *j*, which should be classified as PD patients (*y*_*j*_ = −1) or healthy individuals (*y*_*j*_ = +1). Then, the SVM problem is represented by the following equation:

(2) $$\begin{array}{c} f\left(\text{x}\right)=w.\phi \left(x\right)+b, \end{array}$$

where *w* and *b* indicate the regression coefficients vector (weight) and the intercept (bias) term, respectively. Considering the *ε*-insensitivity loss function for the above equation, the subsequent constrained optimization problem is then used to obtain the regression coefficients:

(3) $$\begin{array}{c} \frac{1}{2}w^{T}w+C\sum \xi_{j}+C\sum \xi_{j}^{*}, \end{array}$$

with respect to the following restrictions

(4) $$\begin{array}{c} \left\{\begin{array}{c} w^{T}\phi \left(x_{j}\right)+b-y_{j}\leq \varepsilon +C\xi_{j},\\ y_{j}-w^{T}\phi \left(x_{j}\right)-b\leq \varepsilon +C\xi_{j}^{*}, \end{array}\right. \end{array}$$

where *ξ*_*j*_^∗^ and *ξ*_*j*_ are known as nonnegative slack variables that are used for penalizing the training errors with the error tolerance of *ε*, and *C* > 0 is the tuning parameter (known as capacity) to determine the empirical error's degree. Finally, the following Lagrange function is used to optimize the problem (3):

(5) $$\begin{array}{c} L\left(w,b,\xi ;\alpha ,\nu \right)=\frac{1}{2}w^{T}w+C\sum \xi_{j}-\sum \alpha_{j}\left\{y_{j}\left(w^{T}\phi \left(x_{j}\right)+b\right)-1+\xi_{j}\right\}-\sum \nu_{j}\xi_{j}. \end{array}$$

In the above equation, the *α*_*j*_ and *ν*_*j*_ are the Lagrange multipliers. Both nonlinear programming tools and a convex quadratic programming problem in *α*_*j*_ can be used to solve the above convex optimization problem. In equation (5), *ϕ* stands for a kernel function (e.g., polynomial, exponential Radial basis, and Gaussian radial basis (GRBF)) that is used to calculate scores for each subject in a nonlinear SVM problem [18, 19].

In the present study, because the sample size was low, a double leave-one-out strategy was used to evaluate the performance of the SVM classifier. To this in each stage of the cross-validation, each time one subject was considered as the testing set and the remaining of them were considered as the training set. In the training set, again, a leave-one-out strategy was used to tune the parameters, and the three tuning parameters were optimized using trial and error over a sequence of values. Also, the radial-based kernel function was used.

### 2.5. Validation of Genes

An external evaluation of the selected genes was conducted using a new data-series GSE54536 with platform GPL10558. Then, this new dataset was considered as testing set, and we predicted the true class of the subjects. The data included 5 PD and 5 normal subjects.

### 2.6. Evaluation Criteria

The accuracy of the diagnostic gene biomarkers for PD was evaluated, and the best ones were found. In this study, the area under the ROC curve (AUC) and the total accuracy were used to assess the models' performance.

(6) $$\begin{array}{c} \text{Accuracy}=\frac{\text{True positive}+\text{True negative}}{\text{total sample}}. \end{array}$$

Analyses were performed by using the R.4.1.0 software programming.

## 3. Result

Based on GSE22491, a total of 41000 differential genes were discovered with 1491 genes being significantly selected (*p* < 0.05, −1 ≤ fold − change ≥ 1) when comparing PD to a healthy control group.

### 3.1. Functional and Pathway Enrichment Analysis

**Figure 2:**
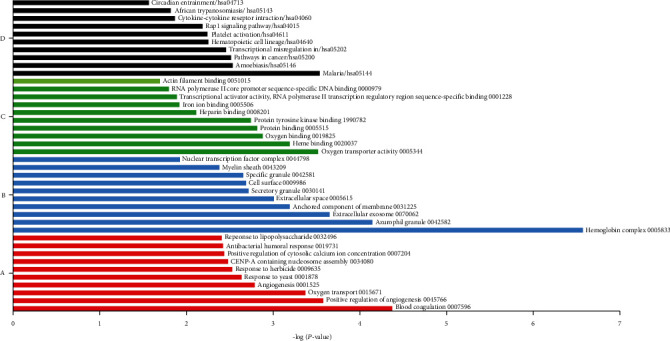
Summary of the top GO and KEGG results for DEGs. (a) Biological process for DEGs. (b) Cellular component for DEGs. (c) Molecular function for DEGs. (d) KEGG pathway for DEGs.

The list of differential genes was entered into the DAVID software for the recognized DEGs. Analyses of GO and KEGG pathway enrichment were utilized, and Fisher's exact test was used to evaluate the results. The 0.05 cut-off *p* value was used for further analysis. According to the findings of the GO study, biological processes (BP), cell component (CC), and molecular function (MF) were all associated with 124, 43, and 31 GO terms.  Figure 2 displays the top 10 GO terms from the GO enrichment and KEGG pathway analysis. In the CC group, the DEGs were significantly enriched in hemoglobin complex, azurophil granule, and extracellular exosome. The DEGs were significantly enriched in oxygen transporter activity, heme binding, and oxygen binding according to the GO molecular function (MF). The DEGs in the BP group are predominantly enriched in blood coagulation, positive regulation of angiogenesis, and oxygen transport. KEGG pathway analysis yielded 19 enriched pathways in malaria, amoebiasis, and pathway in cancer.

### 3.2. PPI Network

The PPI network for DEGs was established and composed of 264 nodes and 502 edges. The constructed network was evaluated with the Cytohubba plugin under Cytoscape software to determine the hub genes.

**Figure 3:**
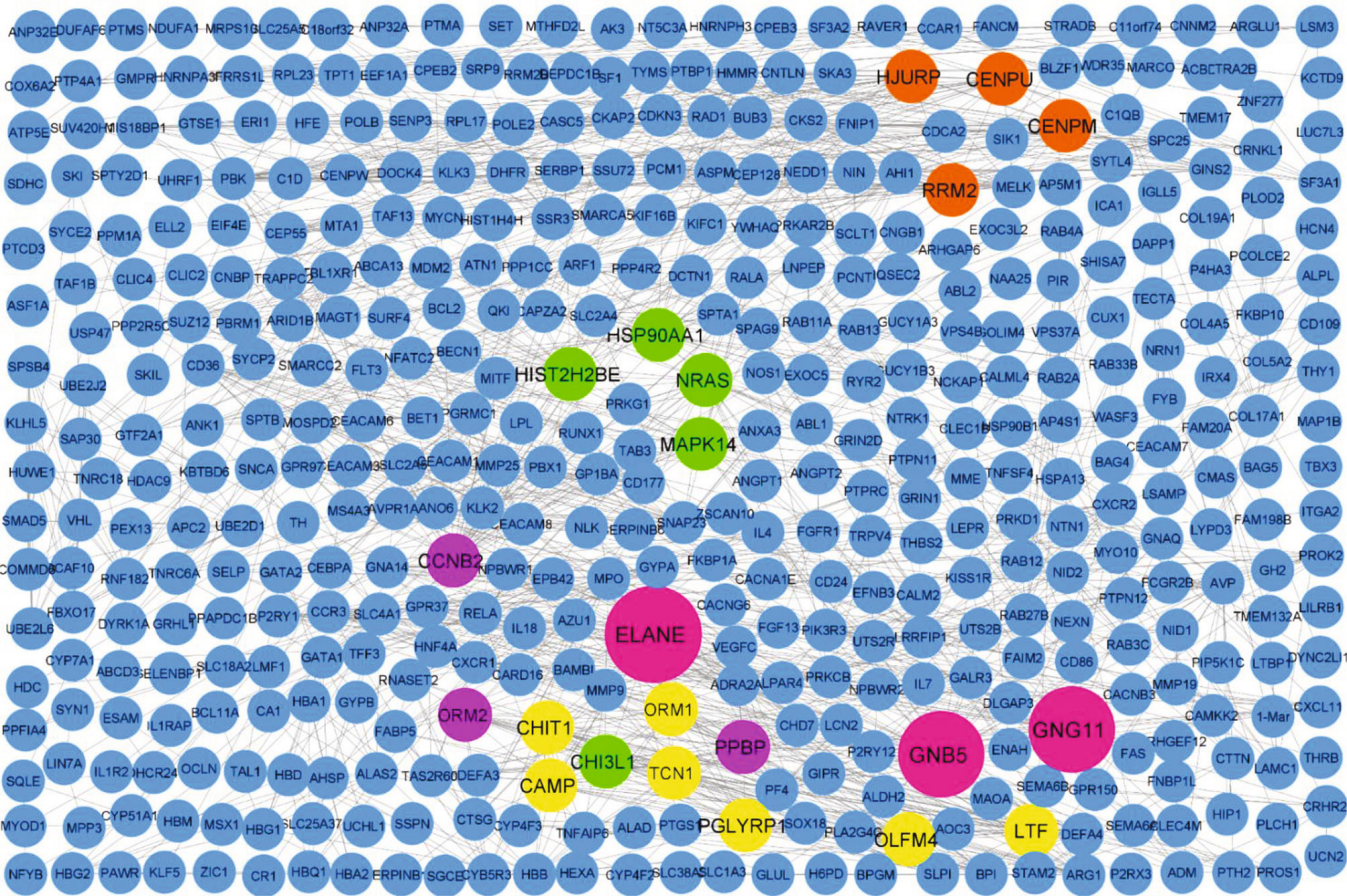
The PPI network of identified DEGs was formed by using Cytoscape software. Proteins are represented by nodes, and interactions between two proteins are described by edges. Orange nodes represent important nodes ranked by the MNC method. Green nodes represent important nodes ranked by the degree method. Yellow nodes represent important nodes ranked by the MCC method. Purple nodes are common proteins between MNC and degree groups. The three big pink nodes were painted as hub genes which are common in three groups.

**Figure 4:**
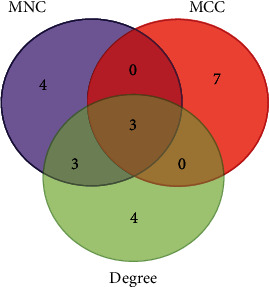
The overlap between the top 10 predicted target genes, ranked by MNC, MCC, and degree, is illustrated in a Venn diagram. The number 3 in the image's center describes the three groups' commonalities.

The top 10 genes ranked by 3 methods including degree comprise (*GNG11*, *GNB5*, *PPBP*, *CCNB2*, *NRAS*, *HIST2H2BE*, *HSP90AA1*, *HSP90AA1*, *MAPK14*, *ORM2*, and *ELANE*), MCC (maximal clique centrality) including (*GNG11*, *GNB5*, *CAMP*, *ORM1*, *CHIT1*, *PGLYRP1*, *OLFM4*, *LTF*, *TCN1*, and *ELANE*), and MNC (maximum neighborhood component) consist of (*GNG11*, *GNB5*, *PPBP*, *CCNB2*, *ORM2*, *ELANE*, *RRM2*, *CENPM*, *CENPU*, and *HJURP*) (Figure 3). Following the advancement of a Venn diagram, 3 common genes were discovered to be hub genes in all three ranking methods (Figure 4).

### 3.3. Statistical Analysis for Determining Sensitivity, Specificity, and Accuracy

An iterative process based on the SVM technique was used to find the relevant genes with the highest sensitivity, specificity, and accuracy. The table below shows the results to fit the support vector machine classification method. The validation method was leave-one-out fitting. The input of the models in the table below was the three genes *ELANE*, *GNB5*, and *GNG11*. Based on the results in the table below, the sensitivity of this method was 0.9 and the specificity of the method was 0.875. According to the overall accuracy criterion, the SVM method had the highest overall accuracy 0.889 in the group classification (Table 1).

We also applied the SVM method to validate the results of the present study. The area under the ROC curve for classification of new data was 0.851, 0.626, and 0.667 for *ELANE*, *GNB5*, and *GNG11*, respectively.

## 4. Discussion

While it is considered that both hereditary and environmental variables have a role in causing disease, the exact pathogenesis of PD remains unclear. In the current study, a bioinformatics study was performed in order to diagnose the disease. As a result, realizing the disease's molecular mechanism is extremely helpful in the diagnosis and cure of the disease.

In the present study, gene expression data with the series number GSE22491 based on the platform of GPL6480 in the GEO database showed that there were 1491 DEGs in PD samples compared with healthy samples. The PPI network for DEGs was designed in the STRING database. After that, bioinformatics analysis was used by MNC, MCC, and degree tools in Cytoscape software. In addition, highest degree proteins including *ELANE*, *GNB5*, and *GNG11* were discovered as hub nodes in PPI network analysis. The functional and biological interactions between DEGs were investigated using GO and KEGG analyses.

In the present study, the hemoglobin complex was identified to be one of the significant enrichment pathways of DEGs in PD. Network analysis demonstrated 6 genes of DEGs involved in this pathway. According to the Santiago et al.'s meta-analysis study, the collection of iron-metabolizing genes such as *ALAS2* was found in the blood of PD patients [20]. In this paper, pathway and network analysis found enrichment in activities related to the hemoglobin complex. Neurodegeneration has been linked to a disruption in iron homeostasis in PD [21]. In addition, several studies have shown that extracellular exosome was enriched in the CC of GO analysis of the PD [22]. Exosomes, small extracellular vesicles, are now known to be important mediators in neurodegenerative diseases like prion, PD, and Alzheimer's, according to research showing [23]. Exosomes that have a role in spreading *α*-synuclein and *β*-amyloid to the extracellular environment were enriched in GO analysis of PD [23].

A glycosylated heme-enzyme MPO was found in the azurophilic granules of neutrophils and macrophages [24]. Result of current study indicated that azurophil granule is one of the pathways that was enriched in GO analysis.

The pathophysiology of the central nervous system (CNS) has been linked to blood coagulation factors and other proteins that impact or are controlled by the coagulation pathway. The protease-activated receptors (*PARs*) have an important role in the regulatory network pathway, which is activated mainly by activated protein C (*APC*) or thrombin [25]. The blood coagulation pathway has also been linked to neurological diseases such as PD and Alzheimer's [26]. In the study by Eckert et al., it was reported that positive regulation of angiogenesis is associated with AD [27]. Some evidence showed that angiogenesis is connected with X-linked dystonia-parkinsonism syndrome [28]. Infante et al. proposed that genes *HBD*, *HBG1,* and *HBM* significantly enriched in oxygen transport were engaged in diverse aspects of the production of PD [29]. The majority of enriched groups were related to binding, such as heme binding, oxygen binding, and protein binding, according to the molecular function of the GO analysis. Oxygen transporter activity was the most significant enrichment of the molecular functions. Andez et al. demonstrated that oxygen transporter activity is associated with AD [30]. In addition, Santiago et al. found that oxygen binding and heme-binding pathways were enriched in MF of PD [20]. In this research, malaria, amoebiasis, and cancer pathways were identified as the most enriched pathways using KEGG pathway analysis.

Elastases are a form of a serine protease that hydrolyze a wide range of proteins, including elastin. Six elastase genes exist in humans, all of which encode structurally related proteins. To make the active protease, the encoded preproprotein is proteolytically processed. This protease hydrolyzes proteins inside specialized neutrophil lysosomes known as azurophil granule lysosomes after activation as well as extracellular matrix proteins [31, 32]. Neutrophil elastase (ELANE) leads to degradation of extracellular matrix components such as elastin, collagen types I–IV, and fibronectin and plays a role in the control of inflammatory response [33, 34]. More ever, ELANE has a role in calcium homeostasis, Gram-negative antibacterial, negative regulation of chemotaxis and inflammation, a decrease of chemokine production, and increasing the IL-8 production and MAP kinase activity [35].

The *GNB5* gene encodes the G protein *β*5 [13, 14]. G*β*5 is part of a signal-transducing G-protein *β* subunit family. G*β*5 is preferentially expressed in the brain and the nervous system, and it is unique in its ability to heterodimerize with G-protein signal-regulating protein family R7 [15, 16]. After binding to the R7 proteins, G*β*5 forms a complex with SNARE-like membrane-anchoring proteins [17]. From work in animal models, we know that the G*β*5 protein is required for normal development of the brain and the retinal photoreceptor layer [2, 3, 14].

The GNB5 which encodes the beta 5 subunit of heterotrimeric G proteins, is predominantly expressed in the nervous system and is essential for natural brain development [36, 37]. One study showed that *GNB5* is a hub gene in Huntington's disease (HD) [38]. Utpala et al. demonstrated that *GNB5* is one of the 10 most significant hub genes involved in GABAergic synapse, Retrograde endocannabinoid signaling, and Ras signaling pathways in AD (Alzheimer disease) [39]. The involvement of *GNB5* in generating attention deficiency hyperactivity disease that is one of the signs of PD is well documented. The central nervous system contains *GNB5*, a *β* component of the GTP-binding proteins. It is known to generate complexes that modulate neuronal transmission activity, impacting behavioral outcomes [40]. According to one report, *GNB5* can be AD-associated (Alzheimer disease) gene [41]. Yalamanchili et al. showed that *GNB5* is one of the top 10 genes in PD patients under treatment [42].

A lipid-anchored plasma membrane protein encoded by *GNG11* belongs to the G protein gamma family [43]. One additional study demonstrated that *GNG11* contributed to the G-protein Coupled Receptor Protein Signaling in AD [44]. Ming et al. showed that *GNG11* was identified as a significant gene in several risk pathways of PD [45].

In a wide variety of applications, the support vector machine (SVM) has proven to be an effective tool that outperforms most other classification systems [46]. In our study, the accuracy of the SVM model was 0.889, with a sensitivity of 0.9 and a specificity of 0.875. In comparison to similar studies, for these three hub genes, the SVM model exhibited the highest accuracy, sensitivity, and specificity so it is recommended that the above genes as diagnostic biomarkers in clinical samples be tested more accurately by molecular methods in future studies. If these experiments are carried out, we might be able to use them as a PD diagnostic biomarker.

## Figures and Tables

**Table 1:** results of fitting support vector machine using ELANE, GNB5, and GNG11 genes.

Method	Sensitivity	Specificity	PPV	NPV	ACC
SVM	0.9	0.875	0.9	0.875	0.889

∗Sensitivity in this table means that 90% of genes were correctly predicted as a causative agent of the disease and specificity means that 87% of genes outside this group do not play a role in causing the disease.

## Data Availability

The expression profile data used to support the findings of this study have been deposited in the GEO database (GSE22491 and GSE54536 ).
